# Intestinal Microbiome in Irritable Bowel Syndrome before and after Gut-Directed Hypnotherapy

**DOI:** 10.3390/ijms19113619

**Published:** 2018-11-16

**Authors:** Johannes Peter, Camille Fournier, Bettina Keip, Nina Rittershaus, Nicola Stephanou-Rieser, Marija Durdevic, Clemens Dejaco, Maria Michalski, Gabriele Moser

**Affiliations:** 1 Division of Gastroenterology and Hepatology, Department of Internal Medicine III, Medical University of Vienna, 1090 Wien, Austria; camille.4nier@gmail.com (C.F.); bettina.keip@meduniwien.ac.at (B.K.); ninarittershaus@gmail.com (N.R.); nicola.stephanou@hotmail.com (N.S.-R.); clemens.dejaco@meduniwien.ac.at (C.D.); maria.michalski@medway.at (M.M.); gabriele.moser@meduniwien.ac.at (G.M.); 2 Center of Medical Research, Medical University Graz, 8036 Graz, Austria; marija.durdevic@medunigraz.at

**Keywords:** intestinal microbiome, irritable bowel syndrome, psychological intervention, hypnosis, psychological stress

## Abstract

Irritable bowel syndrome (IBS) is a disorder with brain-gut-microbiome alterations. Gut-directed hypnotherapy (GHT) has been shown to improve quality of life and symptoms in IBS. This therapy targets psychological coping, central nervous processing and brain-gut interaction. Studies have also demonstrated effects of hypnosis on intestinal transit and the mucosal immune system. So far, no study has examined the effect of GHT on the intestinal microbiome. This study aimed at examining microbial composition, IBS symptoms, and psychological distress before and after GHT. Methods: Fecal samples were collected from 38 IBS patients (Rome-III criteria, mean age 44 years, 27 female, 11 male, 22 diarrhea-dominant, 12 alternating-type and 4 constipation-dominant IBS) before and after 10 weekly group sessions of GHT. Assessments in psychological (perceived stress, PSQ; psychological distress, HADS-D; quality of life, visual analogue scales) and IBS symptom-related variables (IBS severity, IBS-SSS; single symptoms, visual analogue scales) were performed with validated questionnaires. Fecal samples underwent microbial 16S rRNA analyses (regions V1–2). Results: Microbial alpha diversity was stable before and after GHT (chao1 2591 ± 548 vs. 2581 ± 539, *p* = 0.92). No significant differences were found in relative bacterial abundances but trends of reduced abundance of Lachnospiraceae 32.18 (4.14–39.89) Median (Q1–Q3) vs. 28.11 (22.85; 35.55) and Firmicutes: Bacteroidetes ratio after GHT were observable. Significant reductions in symptom severity (323 (266–371) vs. 264 (191–331), *p* = 0.001) and psychological distress 17.0 (12.6–21.8) vs. 12.0 (8.3–18.0), *p* = 0.001, and increased well-being were found after GHT. Adequate relief after therapy was reported by 32 (84%) patients. Conclusion: Reductions in IBS symptoms and psychological burden were observed after gut-directed hypnotherapy, but only small changes were found in intestinal microbiota composition. The findings suggest that hypnosis may act by central nervous impact and other factors largely independent from microbiota composition modulating the brain-gut axis, possibly alterations in vagus nerve functioning and microbiota metabolism.

## 1. Introduction

Irritable bowel syndrome (IBS) is a functional gastrointestinal disorder associated with abdominal pain and altered bowel habits affecting about 11% of the population worldwide [1]. It is currently conceptualized as a disorder of brain-gut interaction [2]. The bacterial microbiome has introduced a new perspective in brain-gut-axis communication and the understanding of IBS [3,4]. Insights from microbiome studies point to bidirectional influences between bacteria, host physiology and behavior [5,6,7], and a role of microbial factors in aberrant gut-brain axis signaling and IBS pathophysiology is strongly hypothesized. However, the evidence for gut microbial alterations in IBS is currently limited [8,9,10]. Alterations in psychobiological stress reactivity are also clearly implicated in IBS [11,12,13]. Data from our own and other research groups have shown associations between psychological stress and microbial factors [14,15,16], and animal models suggest a connection between stress-induced shifts in gut microbiota, visceral pain and altered brain-gut communication [17,18,19]. Presence of psychological distress and comorbidity are also a common clinical feature of IBS [20,21], and it is highly likely that psychological factors contribute to symptom generation and maintenance of the disorder [22,23].

A number of psychological treatments have therefore been developed for IBS, including Cognitive-Behavioral Therapy (CBT), hypnosis and other approaches [24,25]. Gut-directed hypnotherapy (GHT) for IBS was originally developed by Whorwell [26,27]. The documented benefits of GHT comprise direct reduction of IBS symptoms, as well as improvements in quality of life and wellbeing, non-colonic symptoms and decreased anxiety and depression [28,29,30,31]. Despite the clinical success of this therapy, relatively little is known about its pathways of action. Studies have reported evidence for reductions in dysfunctional cognition [32,33], alterations in cerebral pain processing [34], rectal sensitivity [35,36], and change in autonomous nervous system functioning [37].

An involvement of microbiota, or secondary effects of GHT via the brain-gut axis on microbiota seem also plausible for several reasons. Hypnosis elicits a state of relaxation, which is associated with a number of physiological changes, such as reduced heart rate, altered blood flow, metabolism, and brain activity [38,39]. It involves the Hypothalamic-Pituitary-Adrenal axis, which is linked to the gastrointestinal tract by endocrine pathways [40,41,42]. Reductions in systemic and colonic inflammatory factors after GHT were found in patients with ulcerative colitis [43]. Studies reported modulation of gastrointestinal physiology such as orocaecal transit time, gastric acid secretion and gastrocolonic response by hypnosis [44,45,46]. It remains unclear, if or to what extent these mechanisms are relevant for the success of the therapy [47], but they may contribute to microbial effects of hypnosis. Stool consistency and intestinal transit time were identified as markers of microbial composition [48,49], and studies have consistently reported decreases in diarrhea, but also bloating and abdominal pain after hypnotherapy. This leads to the hypothesis of microbial changes after GHT. The aim of this study was to examine the gut microbiome before and after GHT.

## 2. Results

### 2.1. Sample Characteristics

Data from 38 patients with IBS (Rome-III criteria, 27 women and 11 men) were analysed. The mean age in the sample was 44.05 ± 14.48 years (Mean ± Standard Deviation). The majority of patients suffered from diarrhoea-predominant or mixed-type IBS. IBS symptom manifestation (as classified by the IBS-SSS >300 points [50]) was severe in most cases. A post-infectious history of onset (‘PI-IBS’) was present in 7 (18%) patients. Eighteen (48%) were in ongoing additional psychotherapy during the study. Antidepressants were taken by 7 (18%), Mebeverine by 3 (8%) and proton pump inhibitors by two (5%). Domperidone, and Cholestyramine were taken by one patient respectively, and two patients reported the intake of a probiotic supplement. Intake of medication and probiotics was unchanged after GHT.

Baseline sample characteristics are given in Table 1.

### 2.2. Intestinal Microbiome before and after Hypnosis

#### 2.2.1. Alpha Diversity

Alpha diversity before and after GHT was ascertained at a rarefaction depth of 20.000 sequences. There was no significant difference in alpha diversity, *Chao1* amounted to 2591 ± 548 before and 2581 ± 539 (Mean ± Standard Deviation) after therapy (*p* = 0.92, Mann-Whitney *U*-test and Monte-Carlo permutation).

#### 2.2.2. Bacterial Abundance

**Figure 1:**
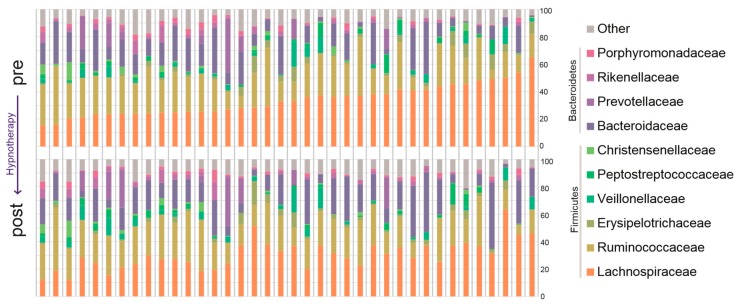
Relative abundance of the ten most prevalent bacterial families before and after gut-directed hypnotherapy (GHT).

**Figure 2:**
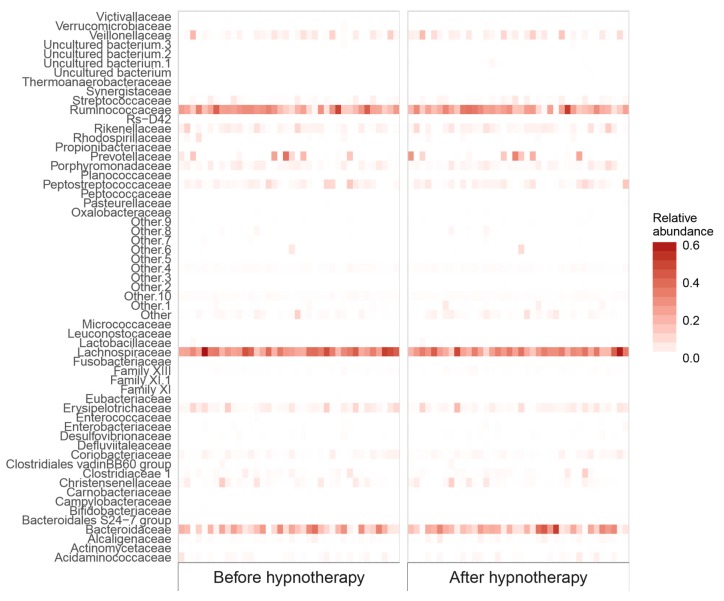
Heatmap of bacterial families before and after GHT.

Bacterial abundances on the family level showed some intraindividual variation before and after GHT, but were altogether largely equal over the two time points. Composition of the ten most abundant families before and after GHT is presented in Figure 1, relative abundances of all 58 bacterial families in the sample are visualized in Figure 2. A heatmap of genus abundances before and after hypnotherapy is provided in Supplementary File 1.

Testing of changes in bacterial abundance after GHT yielded changes in three bacterial families, and in 12 genera (Table 2). However, significances disappeared after controlling for multiple testing.

**Figure 3:**
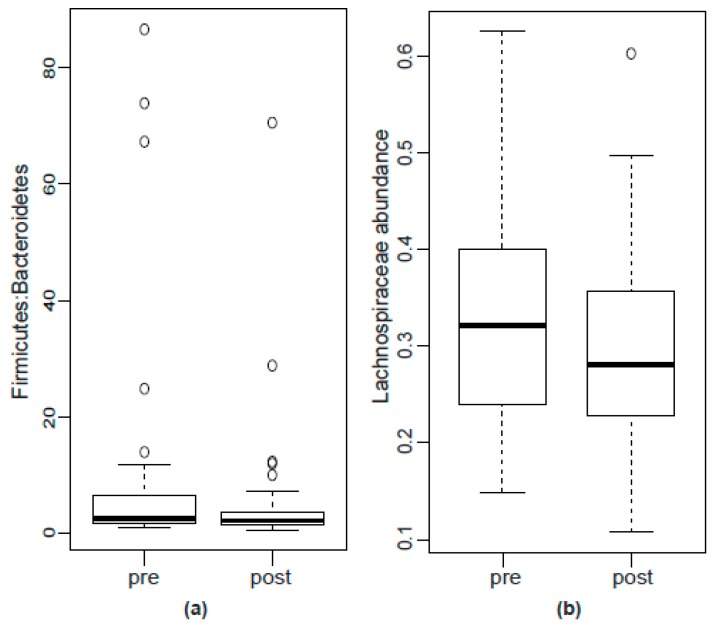
Boxplots of (**a**) *Firmicutes*:*Bacteroidetes* ratio and (**b**) abundance of *Lachnospiraceae* before and after hypnosis. None of the differences were significant.

The most pronounced absolute change in abundance was the reduction in the family *Lachnospiraceae*. In line with this, the *Firmicutes*:*Bacteroidetes* sank from 2.56 (1.77–6.45) before to 2.22 (1.50–3.68) after hypnosis, indicating a slight shift from gram-positives to gram-negative taxa, however the difference was not significant (*p* = 0.34, see Figure 3).

Alterations with *p* < 0.05 were also detected in 161 bacterial OTUs, but none remained significant after FDR correction. One hundred and one (63%) of these OTUs were members of the family *Lachnospiraceae*, and 24 (15%) of *Ruminococcaceae* (Supplementary File 2). With 1.86%, the proportion of OTUs with relevant changes in abundance in comparison to the total number of OTUs seems small even before FDR correction.

### 2.3. IBS Symptoms, Wellbeing and Psychological Stress

**Figure 4:**
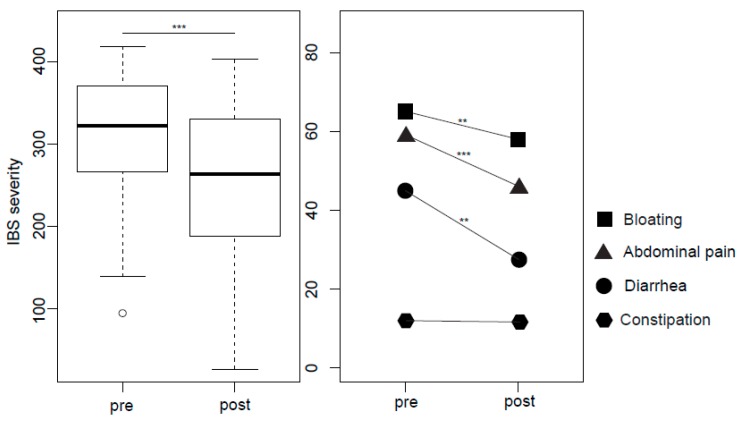
IBS (irritable bowel syndrome) severity (**a**) and IBS single symptoms (**b**) before and after GHT, ** *p* < 0.01, *** *p* < 0.001.

Adequate relief from IBS symptoms after therapy was reported by 32 (84%) patients. Interestingly, all of the seven patients with post-infectious history of onset (PI-IBS) reported adequate relief. Overall, IBS symptom severity decreased significantly after GHT (323 (266–371) Median [Interquartile range] vs. 264 (191–331), *q* < 0.01. Reductions were also present in the IBS single symptoms bloating, abdominal pain, and diarrhea (all *p*’s < 0.001), while constipation remained stable (*p* = 0.19, Figure 4).

**Figure 5:**
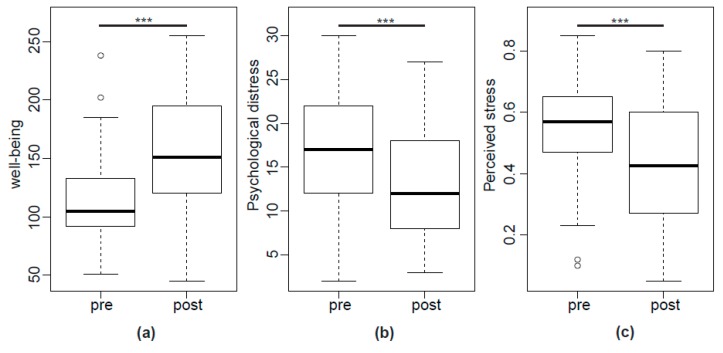
(**a**) well-being (VAS), (**b**) psychological distress (HADS), and (**c**) perceived stress before and after GHT, *** *p* < 0.001.

Well-being (Visual analogue scales pertaining to general, physical and psychological well-being) increased from 105 (92–133) to 151 (122–194), while psychological distress decreased from 17 (12.6–21.8) points to 12 (8.3–18.0) points and stress perception fell from 0.570 (0.478–0.643) to 0.425 (0.278–0.600), all *p*’s < 0.01, see Figure 5.

### 2.4. Relationship between Clinical Improvement and Microbial Changes

**Figure 6:**
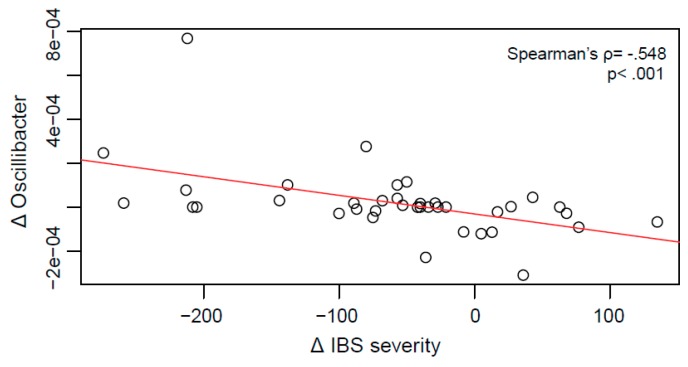
Scatterplot of the relation between changes in IBS severity and changes in abundance of the genus *Oscillibacter* pre-post GHT.

To detect microbial shifts possibly going along with the clinical improvements observed, changes in IBS severity pre-post intervention were correlated with pre-post intervention changes in abundance of bacterial genera. This yielded 17 correlations with Spearman’s rho between 0.32 and 0.55 and *p*-values between 0.049 and *p* < 0.001, however, none remained significant after FDR correction (Supplementary File 3). The strongest association was found in *Oscillibacter*, a member of the *Ruminococcaceae* family (Figure 6).

### 2.5. Microbial Analyses of Sample Subgroups

Since relevant microbial changes may have occurred in subgroups of patients during hypnosis, additional longitudinal analyses were undertaken exclusively in patients who reported adequate relief after therapy (subset of *n* = 32), in patients with IBS with diarrhea (IBS-D, subset of *n* = 22), and in patients with presence of significant psychological distress (subset of *n* = 25). Microbial differences (*p* < 0.05 before controlling for multiple testing) after GHT were present in patients with adequate relief in 11 Genera, 5 families, and one Phylum; in those with IBS-D, in 5 Genera and 3 families; and in patients with psychological distress, in 5 Genera and 2 families. All significances disappeared after controlling for multiple testing. See detailed results in Supplementary File 4. For extensive cross-sectional subgroup analyses (performed with a slightly extended dataset inclusive of the patients analyzed here) see reference [16].

### 2.6. Diet

Complete dietary assessments over one week before and after hypnotherapy were available from *n* = 21 patients, 17 patients had missing data in food frequency questionnaires. Baseline patterns of dietary intake were largely equal to those found in a representative national survey [51] with the exception of a markedly higher consumption of vegetables (exact values in Supplementary File 5). No significant changes, but trends of lower energy intake (−12%), lower consumption of high-caloric foodstuff (−62%) and higher consumption of fruit (+49%), were found after hypnosis. For detailed results on diet see Supplementary File 5.

## 3. Discussion

This study examined the gut microbiome in irritable bowel syndrome before and after gut-directed hypnotherapy. While ground-braking discoveries in the microbiome field have introduced new perspectives in medical and behavioral sciences, and the brain-gut axis is currently a highly influential concept in neurogastroenterology and other disciplines [4,52,53], few studies have assessed the microbiome during or after behavioral interventions. This might be an important approach to elucidate brain-gut-microbiome interactions. To our knowledge, apart from one case study [54], the present study is the first to investigate the gut microbiome during a psychological intervention.

Important limitations of this study were the small sample size, and lack of a control group. Analyses of diet were hampered by incomplete data, and the expressiveness of microbial analyses was severely constrained by loss of power through repeated testing. An additional shortcoming has to be seen in ongoing additional psychotherapies during the study.

The microbial characteristics appeared to remain largely unchanged after the study intervention, gut-directed hypnotherapy. Bacterial diversity remained stable, and none of the changes in abundance were significant after controlling for multiple testing. Relatively consistent trends were observable in reduced *Lachnospiraceae*, one of the generally most abundant families in the human digestive tract [55]. Parallel tendencies of decreases were also present in the *Firmicutes*:*Bacteroidetes* ratio after hypnosis. The tendency of lowered energy intake after hypnosis, that became apparent in nutritional records, might be associated with this observation. A low *Firmicutes*:*Bacteroidetes* ratio was found to be associated with lean phenotypes, younger age, cardiovascular health and a balanced immune system and is generally considered beneficial for health [53,56,57,58]. The present findings are limited to trends, and replication studies are highly required to confirm a possible shift in the *Firmicutes*:*Bacteroidetes* ratio through hypnosis.

The genus *Oscillibacter* showed an association with changes in IBS symptom severity (yet not significant after controlling for multiple testing). Higher gut microbial abundances of *Oscillibacter* were previously identified in humans with depression [59]. *Oscillibacter* produce valeric acid, which resembles GABA and has been shown to bind the GABAa receptor [60,61]. An interaction of the metabolic products of *Oscillibacter* with the enteric and central nervous systems seems therefore plausible and deserves further investigation.

Overall, when examined on higher resolution levels (such as genus, or OTU level), the microbial differences observed between samples pre-post hypnosis seemed relatively unsystematic when taking into account the sheer number of comparisons/variables, and rather caused by random fluctuations than by distinct brain-to-gut modulations. This stands in contrast to the reported symptom improvements, especially reductions in diarrhea. These led to the hypothesis of microbial alterations, as stool consistency is regarded as a proxy for colon transit time, and has shown strong associations with microbiome composition and richness [62]. The majority of patients experienced marked reductions in general IBS severity and symptoms, and increased well-being after gut-directed hypnotherapy. Psychological distress and stress perception were reduced in most of the cases. These clinical improvements add to findings from previous studies [28,31]. The contrast between lack of microbial changes and improvements in IBS symptoms and psychological distress suggests that GHT acts on higher levels of the brain-gut axis, including psychological mechanisms [33], and modulation of processing of interoceptive stimuli [34,63,64]. However, further research on the precise mechanisms of therapeutic modulation of the brain-gut microbiota axis by hypnotherapy is highly required. Initial evidence has shown that hypnotherapy alters Autonomous Nervous functioning [37]. In this context, studies focusing on the role of the vagus nerve, which is an integral substrate of the brain-gut-axis and coordinates a number of motor, perceptive, inflammatory and gastrointestinal functions [65,66] seem promising. With regard to the microbiome, given that in our study microbiota composition was largely unchanged by GHT, possible modulation of the microbiota *function* cannot be ruled out. Metabolomic studies are necessary to elucidate this aspect of therapeutic modulation of the brain-gut-microbiota axis.

## 4. Materials and Methods

### 4.1. Recruitment

The study was conducted at the University Hospital of Vienna, Outpatient Clinic for Psychosomatics at the Department of Gastroenterology and Hepatology, University Clinic of Internal Medicine III. It included patients with IBS diagnosed according to Rome III criteria, aged between 18 and 89, and refractory to other IBS therapies. Exclusion criteria were pregnancy, bowel surgery, mental retardation, insufficient knowledge of German, concomitant severe organic disease or Schizophrenia, Psychosis, Substance-Related Disorder or Panic Disorder, and antibiotic treatment within the month before stool collection. The time for patients to reach the hospital was not to be longer than 1 h. Assessments were performed before and after gut-directed Hypnotherapy. Sixty-three patients were screened for eligibility, 53 were enrolled, 48 were included into cross-sectional analyses [16], and 45 participated in hypnosis. *N* = 38 were included in the present analyses (see flowchart, Supplementary File 6). The study protocol was approved by the ethics committee of the Medical University of Vienna (ID: 1502/2014, (01/08/2014)) and registered (clinicaltrials.gov Identifier: NCT02536131). Informed consent was given by each participant. No financial or other incentives were offered for study participation.

### 4.2. Intervention

Gut-directed hypnotherapy was administered by GM and MM according to the Manchester protocol [27] in 10 weekly sessions (45 min) in groups with 5–7 patients over a treatment period of 12 weeks at the university Hospital as in a previous study [28]. At the first session, participants were informed about the effects of hypnosis, and a compact disc (created by the therapist) was handed out at the third session for practicing at home on a daily basis (practicing was documented). Patients were asked not to change medication, intake of nutritional supplements and diet, and ongoing external psychotherapy during the study.

### 4.3. 16S rRNA Sequencing

Stool samples were collected and frozen by patients, brought to the hospital and deep-frozen at −80 °C. DNA isolation, library preparation, and sequencing were then performed at the Graz University Center for Medical Research as described in Klymiuk et al., 2016 (82). Frozen stool samples were used for total DNA isolation by combination of mechanical and enzymatic lysis with the MagnaPure LC DNA Isolation Kit III (Bacteria, Fungi; Roche, Mannheim, Germany) according to manufacturer’s instructions. Samples were bead-beaten for mechanical lysis at 6500 rpm for 30 s twice in a MagNA Lyser (Roche). After incubation with lysozyme and Proteinase K, enzymes were deactivated at 95 °C for 10 min and DNA purification was performed according to kit instructions. PCR amplification was performed with the target specific primers 27f and 357r and 2 µL of total DNA extract were used for a 25 µL PCR reaction in triplicates containing 1 × Fast Start High Fidelity Buffer, 1.25 U High Fidelity Enzyme, 200 µM dNTPs, 0.4 µM bar-coded primers and PCR-grade water (Roche). The final library was quantified using a Quantus Fluorometer (Promega, Mannheim, Germany) and loaded to an Agilent 2100 Bioanalyzer (Agilent Technologies, Waldbronn, Germany) using a high-sensitivity DNA assay according to manufacturer’s instructions for quality control. A 6 pM library run was performed on a MiSeqII desktop sequencer (Illumina, Eindhoven, The Netherlands) with 20% PhiX control DNA. MiSeq paired-end raw sequence forward and reverse reads were subsequently merged using ea-utils v1.1.2 with standard settings, followed by a split library step from QIIME v1.9.1 and removal sequence reads shorter than 200 nucleotides, reads that contained ambiguous bases or reads with an average quality score of less than 30. Chimera were removed using USEARCH v6. against 97% clustered SILVA reference database (83). Operational taxonomic units (OTUs) were picked utilizing the QIIME open-reference pipeline to perform clustering steps at 97% sequence similarity, the taxonomy assignment with a UCLUST algorithm, alignment of reference sequences with pyNAST and generation of a phylogenetic tree with FastTree.

After filtering OTUs with less than 20 total counts and presence in less than 5 samples, the OTU table contained 8647 bacterial OTUs.

### 4.4. Alpha Diversity Analyses

Alpha diversity was analyzed at a rarefaction depth of 20,000 sequences using the observed species, Faith’s phylogenetic diversity (PD) and estimated richness (chao1) metrics. Alpha comparisons among subgroups were performed by Mann-Whitney *U*-tests and 999 Monte Carlo permutations.

### 4.5. Analyses of Bacterial Abundance

Paired Wilcoxon tests of bacterial abundances were performed on Genus, Family, and Phylum level. Correction for multiple testing was by Benjamini Hochberg’s False Discovery Rate (FDR). 

### 4.6. Correlational Analyses

Spearman correlations were calculated to test associations between change in relative abundance of microbial genera (Δ abundance = post-intervention abundance − pre-intervention abundance) and change in IBS severity (Scores of the IBS-SSS, Δ IBS severity = post-intervention severity − pre-intervention severity).

### 4.7. Questionnaires

IBS severity was assessed with the Irritable Bowel Syndrome—Severity Scoring System (IBS-SSS) [50], a questionnaire for clinical assessment of IBS symptom burden and severity. Values range between 0 and 500, with higher values representing higher symptom burden. Values were classified as mild (values ranging between 75–175), moderate (175–300), and severe IBS (300–500) as proposed. Sound reproducibility, sensitivity and specificity are reported for the German version [67], and the scale has been recommended repeatedly for assessment of IBS in methodological reviews [68,69].

Wellbeing was assessed by single analogue scales pertaining to general, physical and psychological wellbeing as in a former study [28]. The three scales (0 = very bad, up to 100 = excellent) were combined as a measure of wellbeing (0–300), Cronbach’s alpha is 0.96.

Psychological distress was assessed with the Hospital Anxiety and Depression Scale (German version, HADS-D), [70]. The two anxiety and depression scales (each with 7 items and a 4-step response set) are added together as a measure of psychological distress (scores 0–42). Reported internal consistency is Cronbach’s α = 0.80. Presence of clinically relevant psychological distress was defined by depression scores greater than ten, or a combined score of 16 or more [71].

Perceived Stress was measured with the Perceived Stress Questionnaire, German version [72], an instrument assessing subjectively experienced stress independent of a specific and objective occasion with 20 items and 4-step response sets. Cronbach’s α is ≥0.85 for the overall score of the German version. Scores were linear transformed to values between 0 and 1 as appropriate.

The IBS symptoms abdominal pain, bloating, diarrhea, and constipation were assessed by single visual analogue scales (0, not at all present–100, extremely pronounced) as in a previous study [28].

Success of therapy was further asked with a standardized question regarding adequate relief from IBS symptoms (yes/no) [54,68].

### 4.8. Assessment of Diet

Dietary intake was self-recorded by the participants of the study with a food frequency questionnaire (FFQ) over one week and averaged (divided by seven) to obtain the intake per day. Nutritional components were estimated (energy intake in kcal, carbohydrates, lipids, protein per day), and intake of food was summed up into categories (vegetables, fruit, cereals and dairy products, milk products, fish and foods containing unsaturated fats, and high-caloric food: Foods rich in fat and sugar, such as confectionery, chips, chocolate). Only complete FFQs over one week before and after GHT were included for analysis.

### 4.9. Statistical Analyses

Statistical analyses were conducted using QIIME [73], the bioinformatics platform Galaxy [74], and R [75]. Parameter-free testing was adopted as assumptions of normal distribution were violated. *p*-values were corrected by Bonferroni’s method as default in QIIME or otherwise corrected by Benjamini & Hochberg’s method to control for the false discovery rate (FDR) and given as q values. The alpha level was set at 0.05 (two-sided) throughout all tests.

## Figures and Tables

**Table 1:** Baseline sample characteristics.

IBS-D	22 (58%)
IBS-mix	12 (32%)
IBS-C	4 (10%)
Post-infectious IBS	7 (18%)
mild/moderate/severe IBS	1 (2%)/12 (32%)/25 (66%)
Disease duration	7 (3.38–14.25)
Presence of psychological distress	25 (66%)

Frequencies and percentages, or Median (Interquartile range).

**Table 2:** Taxa with altered abundance before and after GHT.

Taxonomy	Before GHT	After GHT	*p*	*q*
Families				
Other Bacteroidetes	0.0465 (0.0092–0.1553)	0.0771 (0.0205–0.2472)	0.0035	0.154
Clostridiales XI	0 (0–0)	0 (0–0.0024)	0.0053	0.154
*Lachnospiraceae*	32.18 (24.14–39.89)	28.11 (22.85–35.55)	0.0199	0.384
Genera				
Other Bacteroidetes	0.0474 (0.0093–0.1725)	0.0568 (0.0205–0.2211)	0.0045	0.549
*Coprococcus 3*	0.0359 (0.0118–0.1134)	0.0310 (0.0102–0.0699)	0.0057	0.549
Uc Lachnospiraceae	0.8217 (0.4973–1.2215)	0.8329 (0.4190–1.1993)	0.0149	0.743
*Clostridiales vadinBB60 group*	0 (0–0.0074)	0.0016 (0–0.0094)	0.0156	0.743
Other Lachnospiraceae	7.3539 (5.0824–10.7166)	6.5448 (4.16041–9.1448)	0.0263	0.743
Lachnospiraceae UCG9	0 (0–0.0090)	0 (0–0.0033)	0.0328	0.743
*Intestinimonas*	0 (0–0.0032)	0.0014 (0–0.0059)	0.0358	0.743
*Anaerofustis*	0 (0–0)	0 (0–0)	0.0376	0.743
Lachnospiraceae UCG10	0.0049 (0–0.0251)	0.0052 (0–0.0142)	0.0385	0.743
*Blautia*	5.3194 (3.4527–7.3214)	4.6071 (2.6657–7.6441)	0.0445	0.743
*Coprococcus 2*	0 (0–0.0016)	0 (0–0.0109)	0.0458	0.743
*Eubacterium ventriosum group*	0.1553 (0.0559–0.4382)	0.1336 (0.0344–0.2815)	0.0464	0.743

GHT: gut-directed hypnotherapy; relative abundances of taxa with changes *p* < 0.05 in percent, Median [Q1–Q3]. *q*-values are FDR-corrected *p*-values.

## Supplementary Materials

Supplementary materials can be found at http://www.mdpi.com/1422-0067/19/11/3619/s1.

## Abbreviations

IBS	Irritable bowel syndrome
GHT	Gut-directed hypnotherapy
FDR	False Discovery Rate
OTU	Operational Taxonomic Unit
HADS	Hospital Anxiety and Depression Scale
VAS	Visual Analogue Scales
GABA	Gamma-aminobutyric acid
