# Supplementary material for: Intestinal Microbiome in Irritable Bowel Syndrome before and after Gut-Directed Hypnotherapy
Source: Int J Mol Sci. 2018 Nov 16;19(11):3619. doi: 10.3390/ijms19113619 (PMC6274728; doi:10.3390/ijms19113619)
Supplement: Supplementary file 1 [file ijms-19-03619-s001.zip › Supplement 4.pdf]

Supplementary File 4. Microbial subgroup analyses.

Subgroup analyses of microbial abundance before and after GHT in patients with adequate relief (n= 32, Table 1), in patients with IBS-D (n= 22, Table 2), and in patients with psychological distress (n= 25, Table 3).

Table 1. Subgroup analysis of abundance before and after GHT in patients with adequate relief (n=32)

Taxonomy	*p*	*q*
Phylum		
Cyanobacteria	0.042	0.815
Family		
Other Bacteroidetes	0.011	0.815
Eubacteriaceae	0.014	0.815
Clostridiales Family XI	0.021	0.815
Lachnospiraceae	0.031	0.815
Uncultured Gastranaerophilales	0.042	0.815
Genus		
Coprococcus 3	0.004	0.815
Uncultured Lachnospiraceae	0.013	0.815
Other Bacteroidetes	0.015	0.815
Anaerofilum	0.020	0.815
Uncultured Ruminococcaceae 9	0.025	0.815
Coprococcus 2	0.033	0.815
Uncultured Gastranaerophilales	0.042	0.815
Eubacterium ventriosum group	0.043	0.815
Ruminococcus gauvreauii group	0.043	0.815
Other Lachnospiraceae	0.043	0.815
Uncultured Lachnospiraceae 10	0.050	0.815

Relative abundances of taxa with changes p<0.05 in percent, Median [Q1-Q3]. q-values are FDR-corrected *p*-values.

Table 2. Subgroup analysis of abundance before and after GHT in patients with IBS-D (n=22)

Taxonomy	*p*	*q*
Family		
Clostridiales Family XI	0.030	1
Veillonellacaeae	0.043	1
Genus		
Anaerofilum	0.014	1
Lachnospiraceae UCG10	0.016	1
Ruminococcaceae UCG11	0.022	1
Ruminococcus 2	0.024	1
Other Clostridiaceae	0.046	1

Relative abundances of taxa with changes p<0.05 in percent, Median [Q1-Q3]. q-values are FDR-corrected *p*-values.

Table 3. Subgroup analysis of abundance before and after GHT in patients with psychological distress (n= 25).

**Taxonomy**	*p*	*q*
Families		
Veillonellaceae	0.008	0.998
Fusobacteriaceae	0.034	0.998
Genera		
Coprococcus 3	0.012	0.998
Ruminococcaceae UCG009	0.014	0.998
Lachnospiraceae UCG010	0.021	0.998
Anaerofilum	0.024	0.998
Coprococcus 2	0.028	0.998

Relative abundances of taxa with changes $p<0.05$ in percent, Median [Q1-Q3]. q-values are FDR-corrected *p*-values.
