# Supplementary material for: Intestinal Microbiome in Irritable Bowel Syndrome before and after Gut-Directed Hypnotherapy
Source: Int J Mol Sci. 2018 Nov 16;19(11):3619. doi: 10.3390/ijms19113619 (PMC6274728; doi:10.3390/ijms19113619)
Supplement: Supplementary file 1 [file ijms-19-03619-s001.zip › Supplement 5.pdf]

Supplementary File 5. Dietary assessments and analyses.

A subset of n=21 patients completed dietary assessments (food frequency questionnaires) before and after gut-directed hypnotherapy. Values from these 21 IBS patients before hypnotherapy (baseline) were compared to those from 380 reference adults from a representative national survey [1] (Table 1).

Table 1. Intake of nutritional compounds of IBS patients and reference values

Taxonomy	IBS patients (n= 21)	Reference adults (n= 380)
Energy kCal	1911 [1393; 2261]	1974 [1221; 2727]
Carbohydrates E%	43,96 [38,79; 51,26]	45,69 [34,24; 57,14]
Lipids E%	34,61 [30,62; 38,93]	35,89 [27,24; 44,54]
Proteins E%	17,01 [14,47; 19,41]	14,46 [10,23; 18,69]
Fruit g/d	93 [36; 240]	125 [4; 260]
Vegetables g/d	267 [163; 474]	120 [64; 209]
Dairy g/d	95 [56; 152]	120 [44; 234]
Meat g/d	84 [56; 152]	99 [38; 179]

Values are Median [Q1;Q3] of intake per day. MJ= Mega joule, E%= percent of total energy intake, g/d= gram/day.

Dietary intake was compared longitudinally, pre and post hypnotherapy. None of the changes was significant, but with negative relative changes in all macronutrients, overall intake tended to decrease. With regard to single food categories, marked (yet non-significant) relative changes were observed in the categories fruit (+ 49%) and high-caloric foods (-62%).

Table 2. Dietary intake before and after hypnotherapy

Taxonomy	Before GHT	After GHT	p	Relative change
Energy kCal	1911 [1393;2261]	1678 [1289;2017]	0.394	-12%
Carbohydrates g/d	209 [154;255]	181 [121;219]	0.079	-13%
Lipids g/d	71 [55;89]	66 [50;98]	0.821	-8%
Proteins g/d	70 [59;93]	67 [50;86]	0.476	-4%
Fruit g/d	93 [36;240]	139 [42;184]	0.614	+49%
Vegetables g/d	267 [163;474]	225 [159;285]	0.274	-16%
Dairy g/d	95 [56;152]	100 [71;187]	0.795	+5%
Meat g/d	84 [47;191]	96 [55;150]	0.279	+14%
High-caloric	104 [41;200]	40 [10;156]	0.391	-62%
Cereals and legumes	251 [187;275]	204 [137;232]	0.079	-19%
Fish and unsaturated fats	39 [15;67]	39 [9;81]	0.779	0%

Values are Median [Q1;Q3] of intake per day. kCal= , E%= percent of total energy intake, g/d= gram/day, p-values from paired Wilcoxon tests.

1. Elmadfa I, H. V., Wagner K, , *Oesterreichischer Ernaehrungsbericht*. Universität Wien: Wien, 2012.
