# Supplementary figures and images for: Intestinal Microbiome in Irritable Bowel Syndrome before and after Gut-Directed Hypnotherapy
Source: Int J Mol Sci. 2018 Nov 16;19(11):3619. doi: 10.3390/ijms19113619 (PMC6274728; doi:10.3390/ijms19113619)

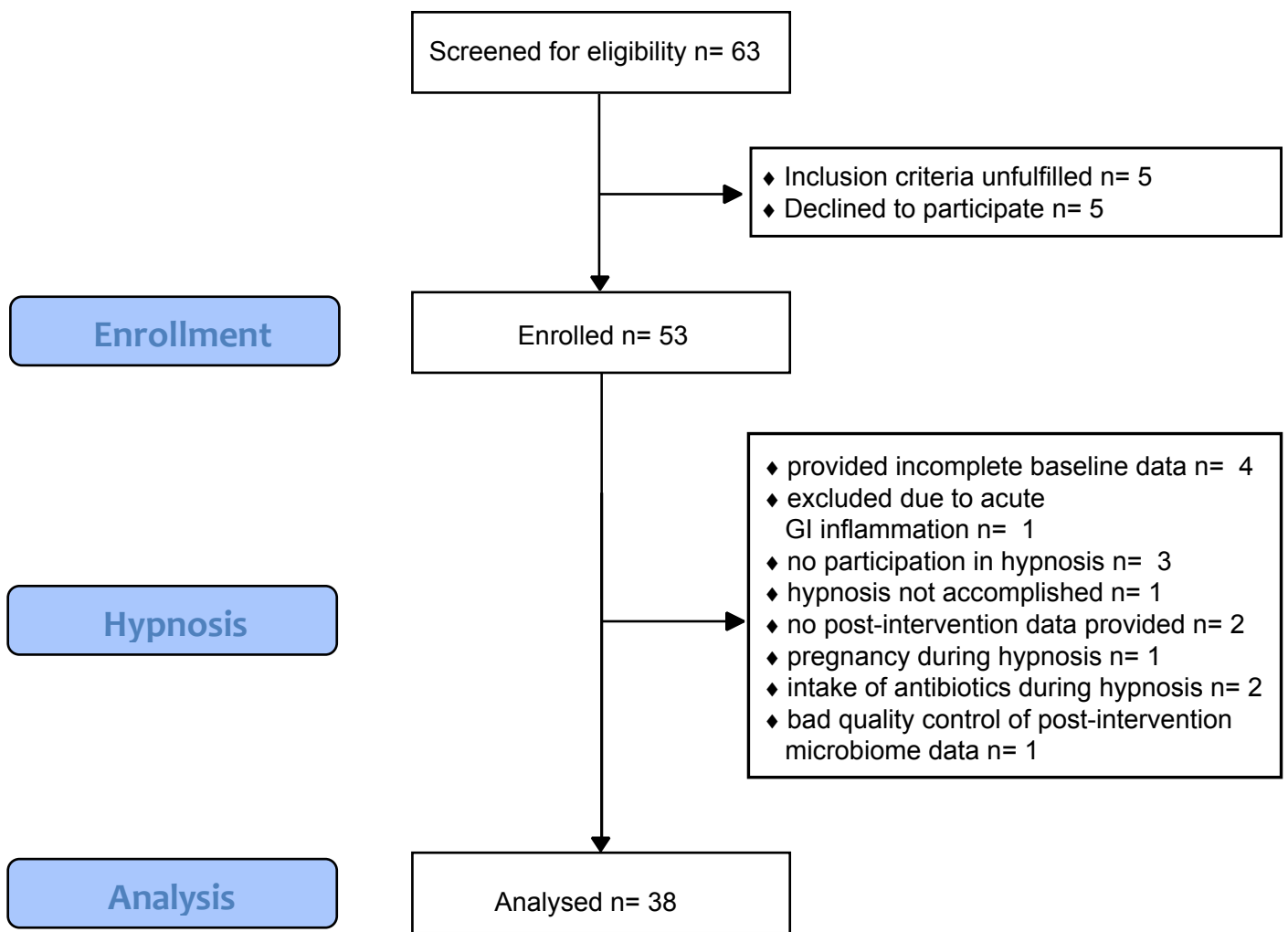

**Figure S6.** Patient Flow Diagram.

Supplement: Supplementary file 1 [file ijms-19-03619-s001.zip › Supplement 6.pdf]
