# Supplementary material for: Intestinal Microbiome in Irritable Bowel Syndrome before and after Gut-Directed Hypnotherapy
Source: Int J Mol Sci. 2018 Nov 16;19(11):3619. doi: 10.3390/ijms19113619 (PMC6274728; doi:10.3390/ijms19113619)
Supplement: Supplementary file 1 [file Supplement 1.pdf › Supplement 1.pdf]

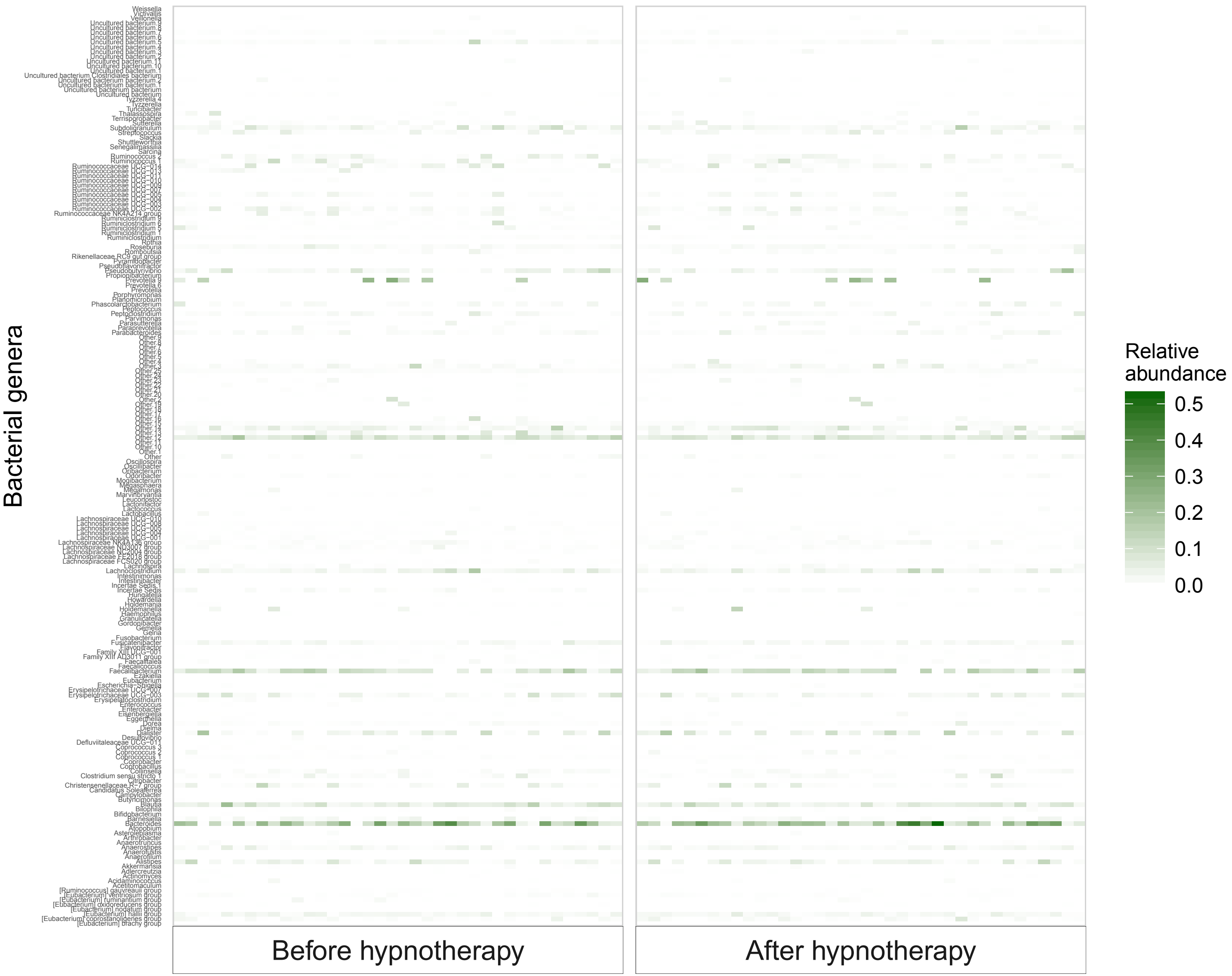

Supplemental Figure 1. Heatmap of abundance of genera before and after hypnotherapy.
